# P105 Sjögren’s syndrome with ANCA-positive multi-system vasculitis with limb-threatening ischaemia

**DOI:** 10.1093/rap/rkag113.135

**Published:** 2026-11-03

**Authors:** Nishita Padmanabhan, Amruth Karthik, Alex Joseph Chandy, Jagdish Ramachandran Nair

**Affiliations:** NHS University Hospitals of Liverpool Group, Liverpool, United Kingdom; NHS University Hospitals of Liverpool Group, Liverpool, United Kingdom; NHS University Hospitals of Liverpool Group, Liverpool, United Kingdom; NHS University Hospitals of Liverpool Group, Liverpool, United Kingdom

## Abstract

**Introduction:**

Sjögren’s syndrome is classically associated with sicca symptoms but is increasingly recognised as a systemic autoimmune condition with extra-glandular involvement, including vasculitis. Overlap presentations can lead to atypical disease patterns, diagnostic uncertainty, and significant morbidity. Vasculitis in Sjögren’s disease is typically small-vessel, and more extensive multi-vessel involvement is less common and may resemble other vasculitis syndromes. These cases are particularly challenging in practice, especially when decisions around immunosuppression are limited by complications such as infection or poor wound healing. We describe a case of suspected Sjögren’s disease presenting with ANCA-positive multi-system vasculitis and limb-threatening ischaemia.

**Case description:**

A middle-aged woman with a prior diagnosis of possible Sjögren’s disease presented with acute-onset progressive ascending weakness, paraesthesia, arthralgia, fever and macular rash. Initial investigations demonstrated MPO-ANCA positivity alongside positive ANA, anti-Ro and anti-La antibodies. She developed vasculitic polyneuropathy with bilateral foot drop. Subsequent nerve conduction studies depicted a mononeuritis multiplex affecting both upper and lower limbs. There was further evidence of systemic involvement, including renal cortical infarcts but with preserved renal function and transient elevation in cardiac biomarkers with a normal cardiac MRI. Her disease progressed rapidly, with distal arterial occlusion in the lower limbs. She was diagnosed with multi-system vasculitis involving small and medium vessels due to tibial artery involvement. Attempts at revascularisation were unsuccessful, and she underwent a right below-knee amputation. Ongoing ischaemia in the left foot led to partial amputations of the hallux and several toes eight weeks later.

She was treated with high-dose corticosteroids initially, iloprost, and 5 cycles of cyclophosphamide. However, immunosuppression was intermittently interrupted due to infection and delayed wound healing. Rituximab is planned but is postponed because of ongoing necrotic wounds and infection risk. She had a drop in eGFR to 50 mL/min/1.73m2, which, when investigated, led to a diagnosis of obstructive uropathy with hydronephrosis due to a ureteric calculus. Results of investigations for renal tubular acidosis came back negative.

Sjögren’s disease diagnosis was supported by ultrasound of the salivary glands demonstrating characteristic changes, despite minimal sicca symptoms and signs. The overall picture suggested overlap between Sjögren disease and ANCA-associated vasculitis, with polyarteritis nodosa also in the differential diagnosis. Despite some stabilisation, she was left with significant morbidity, including limb loss, persistent neuropathy, and recurrent complications related to both disease and treatment.

**Discussion:**

This case highlights a patient with Sjögren’s disease presenting with widespread vasculitic involvement affecting vessels of different sizes. While vasculitis is recognised in Sjögren’s disease, the degree of arterial occlusion and limb-threatening ischaemia seen here is unusual. The diagnosis was not straightforward. Although there had been a prior suspicion of Sjögren’s disease, the absence of prominent sicca symptoms made this less clinically apparent. The presence of MPO-ANCA positivity pointed towards primary ANCA-associated vasculitis such as microscopic polyangiitis, although lung involvement was not seen, while the pattern of arterial involvement raised the possibility of polyarteritis nodosa. This overlap of clinical and serological features made classification difficult and required ongoing reassessment.

The case also reflects the severity of vascular complications in systemic vasculitis. Despite treatment, the patient developed progressive limb ischaemia requiring amputation, alongside neurological involvement and renal infarction.

Management proved challenging. Cyclophosphamide induction had to be interrupted because of infection and poor wound healing. Decisions around escalation to rituximab were delayed due to concerns about worsening infection risk in the context of necrotic tissue. Balancing adequate immunosuppression against treatment-related harm was a recurring issue throughout her course. Overall, this case emphasises that Sjögren’s disease may underlie severe systemic vasculitis and that overlap presentations can evolve over time, requiring flexible and multidisciplinary management.

**Key learning points:**

• Sjögren’s disease can present without sicca symptoms and may present as severe systemic vasculitis.

• Overlap with ANCA-associated vasculitis creates diagnostic uncertainty, particularly with atypical multi-vessel involvement.

• It can be rapidly progressive and limb-threatening despite intensive treatment.

• Management is often constrained by infection and wound healing, requiring careful multidisciplinary balancing of risk and benefit.

